# Characterization of Optimized Ternary PLA/PHB/Organoclay Composites Processed through Fused Filament Fabrication and Injection Molding

**DOI:** 10.3390/ma15093398

**Published:** 2022-05-09

**Authors:** Kubra Buyuksoy-Fekraoui, Clément Lacoste, Monica Francesca Pucci, José-Marie Lopez-Cuesta, Didier Perrin

**Affiliations:** 1 Polymers Composites and Hybrids (PCH), IMT Mines Ales, 30100 Ales, France; kubra.buyuksoy@mines-ales.fr (K.B.-F.); clement.lacoste@mines-ales.fr (C.L.); didier.perrin@mines-ales.fr (D.P.); 2 LMGC, IMT Mines Ales, Université Montpellier, CNRS, 30100 Ales, France; monica.pucci@mines-ales.fr

**Keywords:** PLA/PHB, fused filament fabrication, organo-modified layered silicate

## Abstract

The aim of this study was to investigate the structure–properties relationship of ternary blends of polylactide/polyhydroxybutyrate (PLA/PHB)/organo-modified layered silicate (OMLS). Morphological, thermal, rheological, and mechanical characterizations were performed to understand the influence of OMLS on PLA/PHB (70/30 wt%) formulations optimized through modifications with an epoxy-based chain extender, the use of a plasticizer, as well as the influence of the type of processing route: injection molding or fused filament fabrication. The addition of OMLS allowed the blend compatibility to be improved, with the appearance of a single melting peak on DSC thermograms at 146 °C, as well as the reduction in the size of the nodules for the injected molded specimens. Concerning the printed samples, AFM analysis revealed a coalescence of the PHB minor phase due to its degradation. This phenomenon was dramatically enhanced in the presence of OMLS and has been ascribed to the degradation of both the organo-modifier and the PHB minor phase in the blend. Rheological and mechanical tests (17% decrease in Young’s modulus and 13% decrease in elongation at break) confirmed this degradation that would have occurred during the manufacturing of the filaments and the printing of specimens due to additional thermal and cooling steps.

## 1. Introduction

Polylactide (PLA) is a biobased thermoplastic used in many areas. Its amorphous or semi-crystalline character is governed by the stereochemistry of its polymer backbone. The synthesis of PLA is carried out either by polymerization of lactic acid or lactide [1]. Owing to its physicomechanical properties, PLA is able to replace some oil-based thermoplastics, especially for food packaging applications [2]. One of its main uses is also the processing of filaments for additive manufacturing. However, PLA presents some drawbacks, such as a low level of impact resistance, poor ductility, and slow kinetics of crystallization, which is detrimental to its processing. Moreover, its resistance to hot hydrolysis during processing is relatively poor even if the influence on PLA properties may be limited through the use of chain extenders, particularly based on epoxides, anhydrides, isocyanates, or oxazolines [3,4]. To reduce its brittleness and improve its ductility, PLA can be interestingly combined with other biodegradable polymers [5]. Various polymer blends with PLA have been developed at an academic or commercial scale (PLA/polyhydroxyalkanoate (PHA), PLA/polycaprolactone (PCL), PLA/polybutylene adipate terephthalate (PBAT), etc.). In this article, PLA/PHA blends are investigated due to their biocompatibility and their particular ability to enhance the ductility of PLA [6,7,8].

Layered silicates, more commonly known as clays, possibly organo-modified to produce nanostructures, are commonly used in PLA-based polymer composites [9,10,11]. Organic modifications are the most used methods to improve the properties of layer silicate, allowing a better interaction with polymers. This method allows for increasing the interlayer spacing by intercalation of surfactants or grafting of hydrophobic functional fractions, thus allowing a good dispersion in the polymer matrix to be achieved [12]. Ammonium salts are the most commonly used modifiers [13,14], but other reactive modifiers, such as monomer units, free-radical photopolymerization initiators, and particularly epoxy reagents, are used to cure and reinforce epoxy resins [15]. In the case of the PLA/PHA blend, it is expected that the addition of organo-modified layered silicate (OMLS) in the blend could result in an improvement of Young’s modulus and tensile strength [11,16]. In fact, organo-modified montmorillonite would promote the compatibility between both polymers and limit coalescence processes that could occur during the processing of based-additive manufacturing filaments. Since OMLS has been also used as a compatibilizing agent of PLA blends with other biodegradable polymers such as PBAT, PCL, and poly(butylene succinate-co-butylene adipate) (PBSA) [17], the incorporation of these nanoparticles, whether intercalated or exfoliated, could enable to refine the microstructure by limiting the coalescence of the dispersed phase of nodular morphologies.

The use of PHA in fused filament fabrication (FFF) seems promising, in particular for tissular engineering applications, due to its intrinsic biocompatibility and the possibility to improve the biodegradability and biocompatibility of PLA in PLA/PHA blends [18,19,20]. Previous studies on PLA/PHA blends have shown that the addition of a chain extender (a functionalized styrene–acrylate copolymer with oxirane moieties) could increase the thermal stability of FFF-based 3D-printed specimens [21]. Moreover, epoxy-based chain extenders have been used because the epoxy function can react with hydroxyl and carboxylic acid of polyesters. According to publications in the literature, epoxy-based chain extenders have been used in order to protect PLA and PHA polymers from degradation during the processing, with a good enhancement of mechanical properties [22,23].

Moreover, the incorporation of a plasticizer in such blends (based on the literature) may enhance its ductility. Indeed, the plasticized PLA was used to improve the mechanical properties of the FFF-based 3D-printed specimens [24]. Furthermore, a PLA/PHA blend with the addition of esters from citric acid molecules allowed a higher elongation, from 5% to 187%, at the break of standard specimens to be achieved [25].

Through the optimization of the blend compositions of 3D-printed PLA/PHA blends, a compromise between tensile strength, Young’s modulus, and elongation at break can be achieved.

Hence, the objective of this article is to establish the structure–properties relationship of ternary blends of PLA/PHB/organo-modified clay biocomposites, optimized by the use of a chain extender based on acrylic copolymer with epoxy function and a plasticizer.

A detailed characterization of the microstructures and properties of these complex compositions is performed to understand the interactions between the different components of both FFF-based 3D-printed and injection-molded ternary nanocomposites.

## 2. Materials and Methods

### 2.1. Materials

The blend was composed of a ternary biocomposite based on PLA/PHB/organo-modified nanoclays in which a chain extender based on epoxy functional groups was added. The PLA part was also plasticized. In this section, the entire preparation of the different compositions, as well as the nature of the plasticizer and chain extender used in this blend, are explained in detail.

PLA (Grade 2003D) was supplied in pellet forms by Ingeo^TM^ NatureWorks, Savage, MN, USA, with the following properties: density = 1.24 g/cm^3^ and MFI = 6 g/10 min (210 °C, 2.16 kg). PHB (Grade P209E) was supplied in pellets by Biomer, Schwalbach am Taunus, Germany, with the following properties: density = 1.20 g/cm^3^ and MFI = 10 kJ/m^2^ (180 °C, 2.16 kg). Dibutyl sebacate (DBS) (weight-average molar mass of 314 g/mol) was used as a plasticizer of PLA and supplied by Merck, Darmstadt, Germany. Modified acrylic copolymer with epoxy functions (Joncryl ADR^®^-4468) was obtained from BASF, Germany, with a weight average molar mass of 7250 g/mol and an epoxy equivalent weight of 285 g/mol. Organo-modified clay (montmorillonite with the theoretical formula (OH)_4_Si_8_Al_4_O_20_**·***n*H_2_O) named Byko2block −1200 (BYK) was supplied by BYK Additives and Instruments, Wesel, Germany, with a dry particle size of 50 μm (d50). Cation exchange capacity of montmorillonite and Cloisite 30B are in the 60–100 and 92–95 cmol·kg^−1^ ranges, respectively [26]. The same organic modifier was used for Cloisite 30B and Byko2block-1200, i.e., bis-(2-hydroxyethyl) methyl tallow alkyl quaternary ammonium [27,28].

### 2.2. Blend Preparation

Prior to extrusion, PLA and PHB were dried overnight at 55 °C in a vacuum oven, and Byko2block −1200 was dried overnight at 60 °C. All blends were prepared in a twin-screw extruder BC21 from CLEXTRAL (CLEXTRAL SAS, Firminy, France). The screw diameter was 21 mm, and the extruder length was 900 mm. To limit oxidation and hydrolytic degradation during extrusion, a vacuum pump (Sterling Fluid Systems, Manchester, UK) was used. The processing parameters for all formulations were the following: the screw speed was fixed at 150 rpm, the flow rate was 2.5 kg/h, the temperature of the feed zone was 40 °C, the temperature of the second zone was 120 °C, and the temperature of the other zones and of the die was 180 °C for formulations containing PLA. This temperature is generally used for PLA/plasticizer blends [29,30], whereas a temperature of 165 °C has been used for the PHB/Joncryl ADR^®^-4468 formulation to avoid thermal degradation during processing [31].

**Figure 1:**
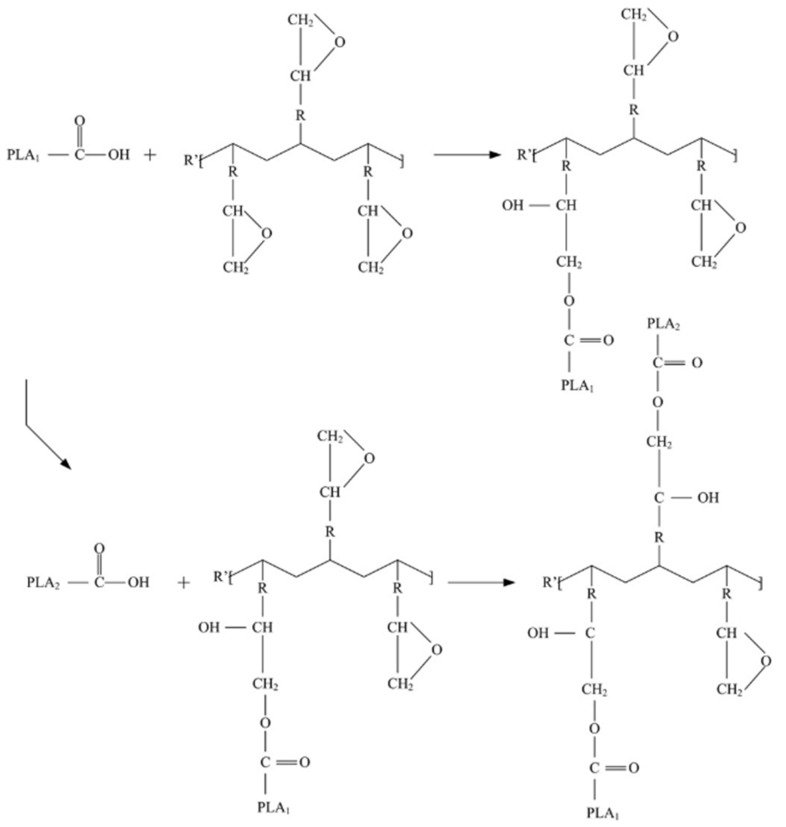
Scheme of PLA-Joncryl reaction to obtain possible long chain branching structures. Reprinted with permission from Ref. [33]. Copyright 2012 Elsevier B.V.

The blends were prepared in several steps. First, reactive extrusion of PLA and PHB blends with 2 wt% and 1 wt% Joncryl ADR^®^-4468 (J), respectively, was performed in order to lengthen the chains of the polymers for better thermal resistance during processing. These formulations were named PLA-J and PHB-J. Theoretically, the epoxy group can react with the hydroxyl and carboxyl groups of the polyesters. The reaction between the carboxyl end groups of polyesters and the epoxide group of chain extenders was demonstrated by Bikiaris and Karayannidis [32]. A scheme of the reaction between carboxyl end groups of PLA and epoxy group of Joncryl (Figure 1) was proposed by Najafi et al. [33], with possible long-chain-branching structures. However, the reactivity between Joncryl and PLA may depend on the processing parameters, since the addition of Joncryl during the processing by twin-screw extrusion can increase the shear stresses due to the increase in the viscosity of the mixture, thus inducing degradation of the polymer [34]. In the case of PLA-J and PHB-J, extrusion parameters, as well as the Joncryl-ADR^®^-4468 rate, were optimized to avoid polymer degradation. Then, PLA-J was plasticized with dibutyl sebacate (DBS) at 5 wt%, to improve its ductility, and was called PLA-JD. Once PLA-JD and PHB-J were obtained, these two polymers were mixed at a ratio of 70/30 (wt%) and extruded to obtain the formulation PLA-JD/PHB-J. The formulation with OMLS was obtained by following the same protocol as the 70/30 formulation, and the 5 wt% organoclay (BYK) was added in the extruder during the mixing of the 70PLA-JD30PHB-J formulation in order to avoid a potential degradation of the polymers. This formulation was named 95(PLA-JD/PHB-J)5BYK. Before each extrusion, the polymers were dried overnight under vacuum at 55 °C. These formulations are summarized in Table 1.

### 2.3. Sample Preparation

The pellets obtained for all the blends were dried under vacuum at 55 °C before the injection molding and the extrusion of calibrated filaments for FFF-based 3D printing.

Calibrated filaments were obtained using a dedicated Yvroud extrusion line (Orléans, France) at 210 °C for PLA-JD/PHB-J blend and at 205 °C for 95(PLA-JD/PHB-J)5BYK formulation. The filament production line was equipped with a single screw extruder (H2528, Mondeo SRL, Montecchio Maggiore, Italy), a cooling bath (BAI-3000, Yvroud, Orléans, France), a crossed laser diameter sensor (ODAC 15XY, Zumbach, Orpund, Zwitzerland), a controlled pulling system (AR03-35, Yvroud, Orléans, France), and a winding tool (CY600-1-SP, Yvroud, Orléans, France). The diameter of the filament was 2.87 ± 0.04 mm. The 3D-printing specimens (1B dog-bone samples) were obtained with an A4v3 FFF printer from 3ntr (Oleggio, Italy), following ISO 527-2 standard and with a 100% filling ratio. The printing direction was +45°/−45° with respect to the sample longitudinal direction. The printing parameters are summarized in Table 2.

The injection molding was performed using a Krauss Maffei KM50-180CX equipment from Krauss Maffei (Munich, Germany) at 210 °C for PLA-JD/PHB-J blend and 205 °C for 95(PLA-JD/PHB-J)5BYK blend to obtain the specimens following ISO 527-2 standard (1A dog-bone samples).

### 2.4. Characterizations

The tensile tests were performed using a universal tensile machine Zwick 7010 (Ulm, Germany). Each test was repeated 5 times using the injected 1A and printed 1B dogbone samples. Young’s modulus was determined at a speed of 1 mm/min. The stress and elongation at rupture were determined at a speed of 50 mm/min for the injected samples and 25 mm/min for the printed samples. Since the sizes of injected and printed samples were different, the speed was divided by two in order to keep the same displacement speed and compare the mechanical properties of the injected and printed specimens.

X-ray diffraction (XRD) analyses were performed on injected and printed dog-bone samples using a Bruker (Champs-sur-Marne, France) X-ray diffractometer, with Cu Kα (λ = 1.54 Å) radiation. A cross-section of the sample was obtained by cryo-fracture at the middle of the dog-bone specimen and placed on the sample holder for analysis.

A scanning electron microscope (SEM) FEI Quanta 200 FEG (Hillsboro, OR, USA), operating at 4 kV, was used to observe the morphology of the blend. The samples were cryo-fractured in the transverse direction and covered with conductive carbon before observation.

For atomic force microscope (AFM) observations, a Leica Microsystems EM UC7 ultra-cryo-microtome was used to prepare the samples in order to obtain a very flat surface. The microstructure of the blends was then observed with the AFM. AFM imaging was obtained using an MFP-3D AFM from Asylum Research in tapping mode. All the samples were tested at a scan rate of 1 Hz with a silicon AFM probe (Asylum Research AC240TS-R3, Neuchâtel, Switzerland, a resonance frequency of 70 kHz, *k* = 2 N·m^−1^), and topography and phase images of 5 × 5 µm^2^ were obtained.

Differential scanning calorimetry (Diamond DSC), from PerkinElmer (Boston, MA, USA), was used to characterize the phase transitions of the blends. The samples for all analyses were obtained from the middle and center of the dog-bone specimens. First, the samples were heated from 30 °C to 190 °C at 10 °C/min in order to remove the thermal history. The samples were then cooled to −50 °C at 5 °C/min and finally heated again at 5 °C/min up to 190 °C. The degree of crystallinity of the different phases in the blend was calculated with the following equation recorded on the second stage of heating [4]:

(1) $$X_{c\;}\left(\%\right)=\left(\frac{H_{m}-H_{c}}{w\times H_{m}^{0}}\right)\times 100\%$$

where $H_{m}$ is the specific melting enthalpy, $H_{c}$ is the cold crystallization enthalpy, w is the weight fraction of the material in the blend, and $H_{m}^{0}$ is the melting enthalpy of a 100% crystalline PLA (93.0 J/g [35]) and PHB (146.0 J/g [36]).

Rheology tests were performed on samples produced by 3D printing with the same printing parameters used for the dog-bone samples. Prior to analysis, each sample was dried under vacuum at 55 °C overnight. Rheological analysis was carried out using a rotational rheometer MCR 702 TwinDrive, Anton Paar, with a parallel plate geometry (2 mm diameter) and a 1.3 mm gap at 180 °C for PLA-JD/PHB-J formulation and 170 °C for 95(PLA-JD/PHB-J)5BYK formulation, under dry nitrogen flow. The linear viscoelastic region was determined with strain sweep at a constant frequency of 1 Hz and 0.5 Hz for the formulation without clay and with clay, respectively. The limit of the linear viscoelasticity region was not determined for the blend containing clay, as thermal degradation was shown by dynamic time sweep tests. The frequency sweep tests were performed at a strain of 0.01% from 126 to 0.06 rad/s.

The thermal stability of OMLS and all blends was investigated using a PerkinElmer Pyris-1 (Perkin Elmer SAS, Evry, France) thermogravimetric analysis (TGA), by varying the mass of the sample during decomposition. The samples were heated under a nitrogen atmosphere, from 30 °C to 900 °C at 10 °C/min.

## 3. Results and Discussion

### 3.1. XRD Behavior

**Figure 2:**
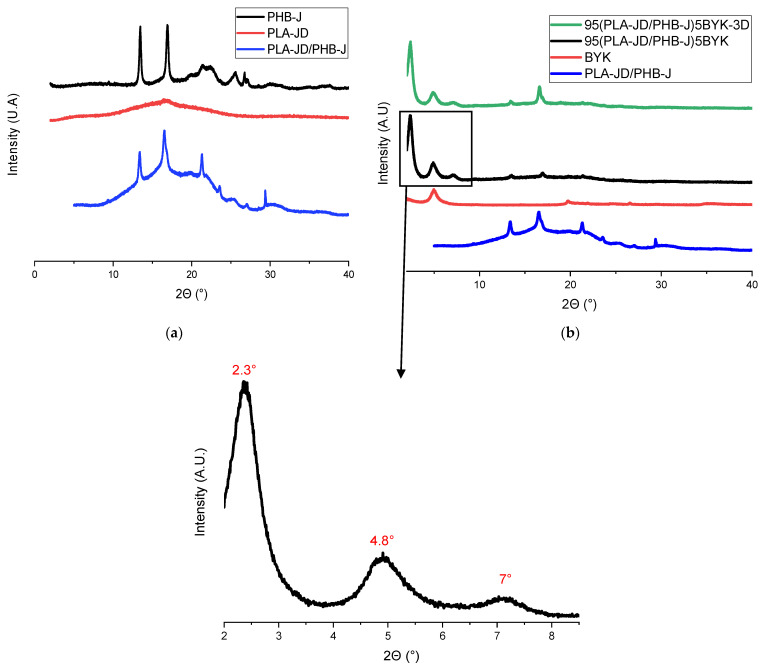
XRD patterns of (**a**) neat PLA-JD, neat PHB-J, and PLA-JD/PHB-J blends processed by injection molding; (**b**) clay and PLA-JD/PHB-J with and without clay processed by injection molding, and 95(PLA-JD/PHB-J)5BYK-3D processed by 3D printing.

Figure 2 shows XRD patterns of neat PLA-JD, neat PHB-J, PLA-JD/PHB-J (Figure 2a), 95(PLA-JD/PHB-J)5BYK blend processed by injection molding, and 95(PLA-JD/PHB-J)5BYK blend processed by 3D printing—called 95(PLA-JD/PHB-J)5BYK-3D (Figure 2b). From the diffractogram of PHB-J, two peaks can be distinguished at 2θ = 13.5° and 16.9°, associated with the (020) and (110) planes of the orthorhombic unit cell of PHB, respectively, whereas the diffractogram of PLA-JD shows an amorphous structure (Figure 2a). The pattern of the PLA-JD/PHB-J blend is similar to that of PHB-J, with a peak at about 2θ = 23°, which could be attributed to the presence of the PLA-JD crystalline phase. This peak indicates that the addition of semi-crystalline PHB-J could improve the crystallinity of PLA-JD because it has been noticed in the diffractogram of fully crystallized PLA, suggesting that PHB acts as a nucleating agent in PLA [7,11].

The main distinct peak present in the OMLS diffractogram is at 2Θ = 4.8° (Figure 2b) with **a** corresponding distance between layers (d001), calculated with Bragg’s law as 1.81 nm. The peak corresponding to the unmodified sodium montmorillonite is located at 2Θ = 7° [37], with d001 = 1.2 nm. The increase in the interlayer distance between the unmodified sodium montmorillonites and the Byko2block-1200 corresponds to the introduction of organic molecules in the interlayer spacing of layered silicate. The interlayer distance (d001 = 1.81 nm) and the thermal degradation profile (see Figure 8 infra) of BYK are similar to those of Cloisite 30B [19]. It is, therefore, suggested that Byko2block-1200 has the same organic modifier as Cloisite 30B, i.e., bis-(2-hydroxyethyl) methyl tallow alkyl quaternary ammonium [27,28]. When the layered silicate is added to the PLA/PHB blend, two additional peaks appear at 2Θ = 2.3° and 2Θ = 7.0° (Figure 2b), which correspond to the interlayer distances of 3.72 nm for the major one and of 1.26 nm for the minor one. The increase in the distance between the layers can be associated with the insertion of polymer chains between the clay platelets, leading to the formation of an intercalated structure [38]. This increase can result from hydrogen bonding interactions between the hydroxyl groups of the organic modifier of the clay and the carbonyl groups of the polyester chains [38,39,40,41]. The other two peaks appearing in the 95(PLA-JD/PHB-J)5BYK blend at 2Θ = 5° and 2Θ = 7° correspond to the non-intercalated clay and to a minor fraction of clay losing its organo-modifier. This means that mainly two types of clay structures co-exist in the blend.

When comparing the diffractograms of 95(PLA-JD/PHB-J)5BYK and 95(PLA-JD/PHB-J)5BYK-3D, there is no difference and the same peaks appear. These results suggest that clay intercalation occurs during twin-screw extrusion and that 3D printing does not influence the nanostructure.

### 3.2. Morphology

**Figure 3:**
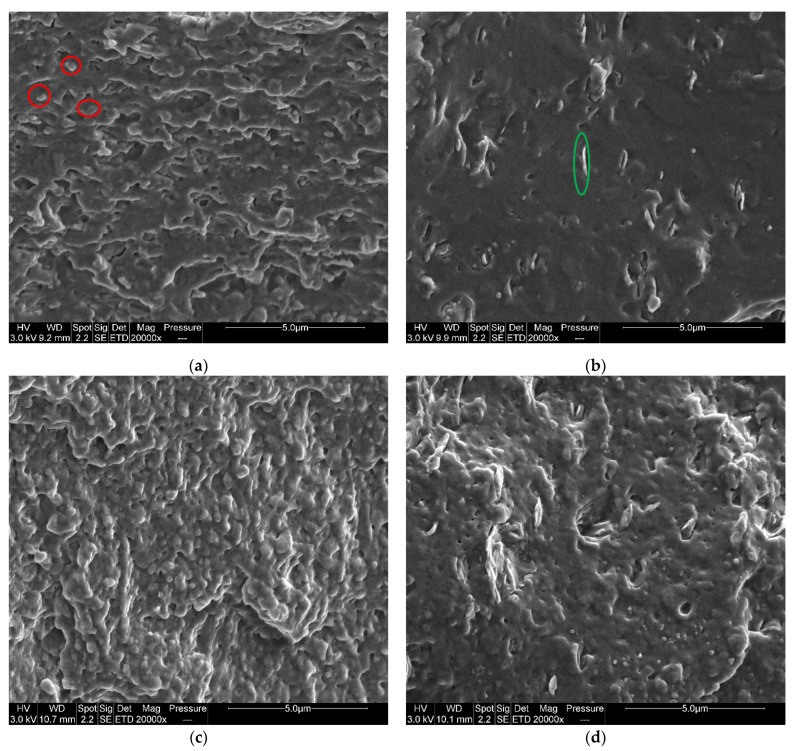
SEM micrographs of transversal cryo-fractured of (**a**) PLA-JD/PHB-J and (**b**) 95(PLA-JD/PHB-J)5BYK injected samples, and (**c**) PLA-JD/PHB-J-3D and (**d**) 95(PLA-JD/PHB-J)5BYK-3D printed samples.

SEM micrographs of PLA-JD/PHB-J and 95(PLA-JD/PHB-J)5BYK are, respectively, shown in Figure 3a,b. In the PLA-JD/PHB-J sample (Figure 3a), the PHB nodules are barely visible, with few cavities corresponding to nodule pull-out. With the addition of OMLS, the fracture surface of the PLA-JD/PHB-J blend becomes sharper.

Organo-modified clays appear in Figure 3b as platelets circled in green. D’Anna et al. [11] have investigated PLA/PHB/nanoclay (Cloisite 5: organo-modified montmorillonite with bis(hydrogenated tallow alkyl) dimethylammonium) blends and, through the determination of the wetting coefficient in a state of thermogravimetric equilibrium, have predicted that the organo-modified clays are mainly located at the interface between PLA and PHB. It can be seen in Figure 3b that PHB nodules seem also preferentially located around the clay platelets and hence at the interface between PLA and PHB. When the specimens are processed through additive manufacturing (Figure 3c,d), the fracture surface appears less sharp, for both filled and unfilled blends.

**Figure 4:**
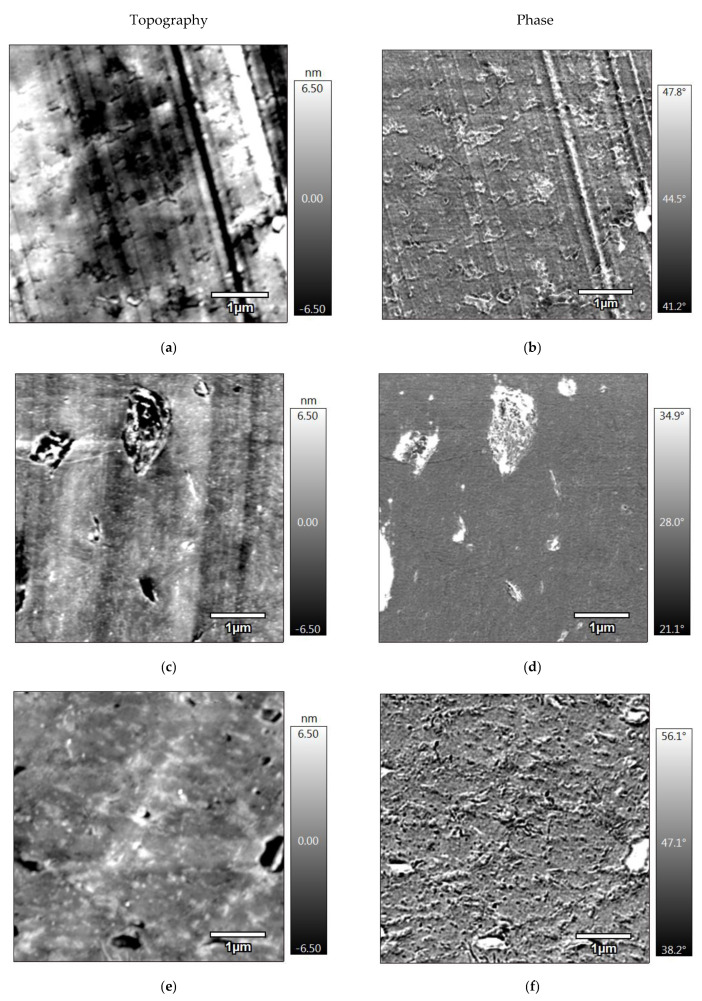

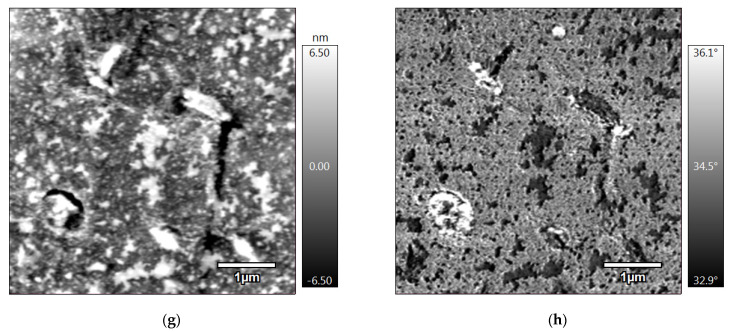
AFM topography and phase images of (**a**,**b**) PLA-JD/PHB-J, (**c**,**d**) 95(PLA-JD/PHB-J)5BYK, (**e**,**f**) PLA-JD/PHB-J-3D, and (**g**,**h**) 95(PLA-JD/PHB-J)5BYK-3D.

The AFM images (Figure 4) complete the observations of blends carried out by SEM observations. Figure 4a–d correspond to the injection-molded blends. The topographic image in Figure 4a and phase image in Figure 4b represent the PLA-JD/PHB-J microstructure, and respective images are shown for the 95(PLA-JD/PHB-J)5BYK (Figure 4c,d).

In Figure 4a, small nodules can be detected that would correspond to the PHB-J phase in the blend. The scratches appearing in the image correspond to knife marks due to the sample preparation of very flat surfaces. These nodules are less visible in the phase image (Figure 4b), but we can see some agglomerates of the minor phase, which could be ascribed to a migration of the plasticizer from the PLA-JD phase. In Figure 4c,d, a drastic decrease in the size of nodules can be observed, which can be attributed to a compatibilization effect of the clay. The compatibilization of polyester blends by the addition of OMLS has already been reported in the literature, according to which a decrease in the size of the nodules was highlighted [17] and related to a mechanism of obstruction of the coalescence for the dispersed phase. Nevertheless, the dispersion of clays is not optimal, possibly due to the presence of non-intercalated clays and some stacks of clay platelets are visible.

Figure 4e,f correspond to PLA-JD/PHB-J and 95(PLA-JD/PHB-J)5BYK processed by 3D printing. It can be observed that the additive manufacturing process does not have a significant influence on the morphology of PLA-JD/PHB-J-3D. We can only note that the aggregates are well visible and seem oriented.

Regarding the 95(PLA-JD/PHB-J)5BYK-3D sample, additive manufacturing entails the formation of clusters containing PHB (visualized in white in Figure 4e and in black in Figure 4f) and distributed in the PLA-JD major phase. Consequently, the compatibilizing effect of OMLS is less effective when the formulation is processed by 3D printing. Nofar et al. [42] studied PLA/PBAT blends and showed that coalescence of the minor phase could occur when a component of the blend is degraded. In our case, the specimens processed through additive manufacturing underwent a different thermomechanical history (enhanced residence time) and additional cooling, compared with injection-molded samples that could generate PHB-J degradation. The polar nature of the organo-modifier could also play a role in these coalescence and agglomeration phenomena. Indeed, it can be assumed that hydrogen bonds between the organo-modifier of the clay and the PHB-J could generate a coalescence of the PHB-J particles. Moreover, even a limited thermal degradation of the polar substituents of the alkylammonium through the Hoffman reaction could cause a hydrolytic degradation of biopolyesters. Thermal degradation of a PLA-based polymer blend containing OMLS due to the degradation of the organo-modifier has previously been highlighted by La Mantia et al. [43].

### 3.3. Mechanical Behavior

**Figure 5:**
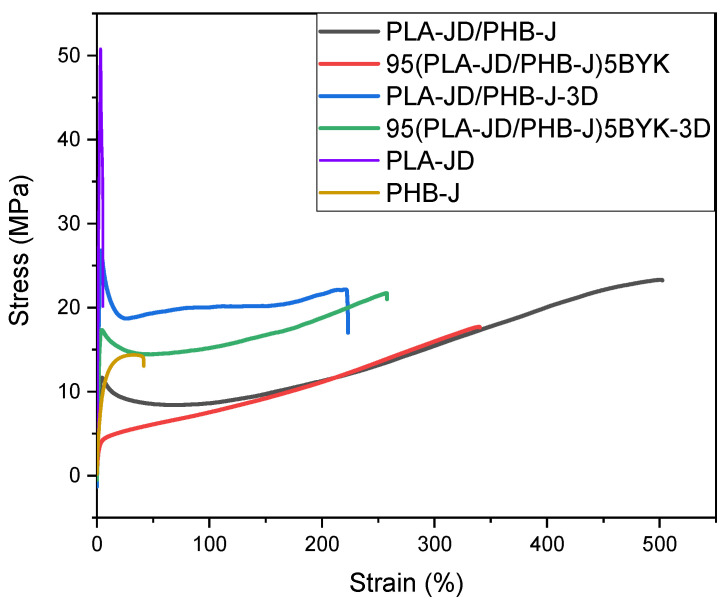
Tensile Stress–strain behavior of injected and printed blends.

**Figure 6:**
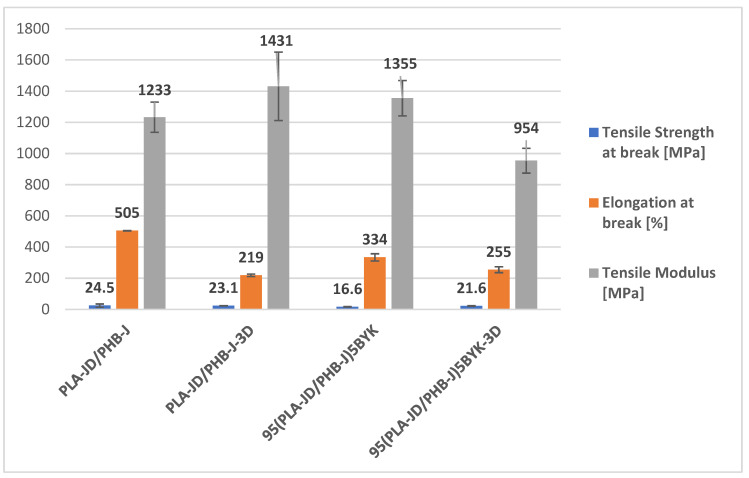
Column graph of tensile strength at break, elongation at break, and tensile modulus for PLA-JD/PHB-J and 95(PLA-JD/PHB-J)5BYK injected and printed blends.

Results of tensile tests for each polymer and its blends are presented in Figure 5, Table 3, and Figure 6. As it can be seen, the deformation profile changes with the addition of OMLS, but also as a function of the processing mode. Although PLA-JD contains a plasticizer, a comparison between the individual polymers and the injection-molded blends shows that blends are more ductile than individual polymers. However, PLA first reacts with Joncryl ADR^®^-4468 to lengthen its chains. This seems to thwart the action of the plasticizer, but the high values of elongation at the break of blends seem to show that the plasticizer is more effective in the blends.

When PLA-JD is blended with PHB-J, this additional extrusion step could allow for a better plasticizer diffusion, resulting in better plasticization, but at the expense of maximal tensile stress and Young’s modulus. Indeed, X. Yang et al. [44] also reported that the addition of PHB to plasticized PLA results in 66% higher elongation.

The presence of OMLS in the blend tends to reduce the elongation at break and the yield stress. Consequently, the compatibilization effect of OMLS and the formation of a nanostructure owing to the intercalation shown by X-Ray diffraction seems to be offset by coalescence phenomena promoted by hydrogen bonding interactions and degradation processes.

The deformation profile corresponding to the sample processed through 3D printing exhibits higher yield stress in comparison with PLA-JD/PHB-J. It can be related to the orientation of the dispersed phase, as observed in AFM images. The increase in tensile properties can also be attributed to the stiffening action of the slightly textured structure formed during ±45° raster angle orientation of 3D printing [45].

Concerning the 95(PLA-JD/PHB-J)5BYK-3D blend, a significant decrease in Young’s modulus, as well as yield stress and elongation at break, is noticed in comparison with 95(PLA-JD/PHB-J)5BYK. This can be ascribed to the strong coalescence or aggregation of PHB-J nodules and their degradation during the processing of the filament in presence of OMLS, as observed in AFM images.

### 3.4. Thermal Properties

#### 3.4.1. DSC

**Figure 7:**
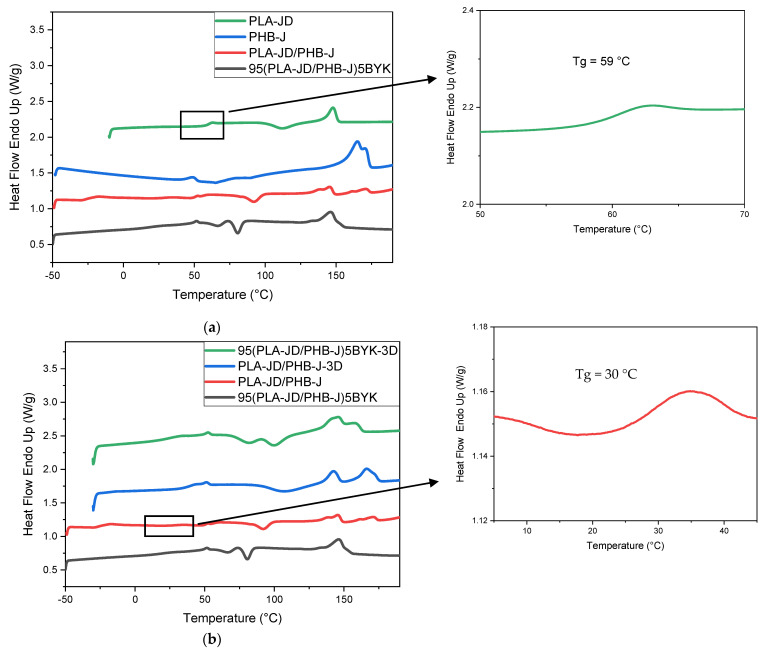
DSC thermograms of (**a**) the sample processed through injection molding and (**b**) a comparison between the samples processed by injection molding vs. 3D printing.

Figure 7 represents the second heating of the samples obtained by DSC analysis, and Table 4 summarizes the results obtained from these thermograms: melting temperature (Tm), glass transition temperature (Tg), cold crystallization temperature (Tcc), and degree of crystallinity (X). PLA-JD shows a single melting peak at 149 °C with a Tg = 59 °C and a cold crystallization peak at 112 °C. PHB-J shows a melting peak at 165 °C with a shoulder. This shoulder may be due to the presence of the chain extender which creates two modes of crystallization, but for PHB-J, no Tg is observed. For the PLA-JD/PHB-J blend, two melting peaks at 146 °C and 171 °C are reported, which can be attributed to the PLA-JD phase and PHB-J phase, respectively. Two Tg at 30 °C and −24 °C are found, which correspond to PLA-JD and PHB-J phases. The decrease in the Tg in the PLA-JD/PHB-J blend is due to a second blending of the modified PLA with the plasticizer, leading to a better diffusion of this sample. This is also noticed for the mechanical properties because of the increase in the elongation at break from 5% to 505%.

The addition of OMLS allows a single melting peak at 146 °C to be obtained, which confirms the compatibilization effect of nanoclay. The decrease in the cold crystallization temperature can be attributed to a nucleation effect of the clay [10]. This effect is reduced when the sample is processed by 3D printing because the Tcc increases. The decrease in the compatibilization effect of OMLS is also observed by the appearance of a second peak when the sample is obtained by 3D printing. These results are due to the degradation of PHB-J during filament processing. An increase in the percentage of crystallinity of PHB-J is noted when it is processed by additive manufacturing. This result can be attributed to the scission of the macromolecules chains caused by the degradation of PHB-J [46].

#### 3.4.2. Thermogravimetric Analysis

**Figure 8:**
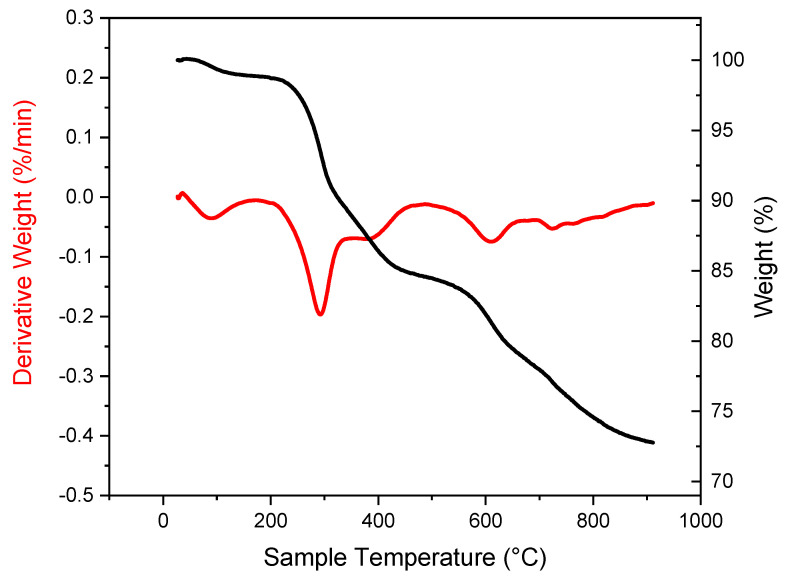
TGA and derivative curves of clay (Byko2block).

Figure 8 represents the weight of OMLS as a function of temperature. The weight loss observed from around 210 °C to around 450 °C is due to the first stage degradation of the organo-modifier of the quaternary alkylammonium used as an organic modifier of the Cloisite 30B [19]. In addition, a second degradation stage starts from 450 °C and would correspond to the de-hydroxylation of the layers of the clay [33].

**Figure 9:**
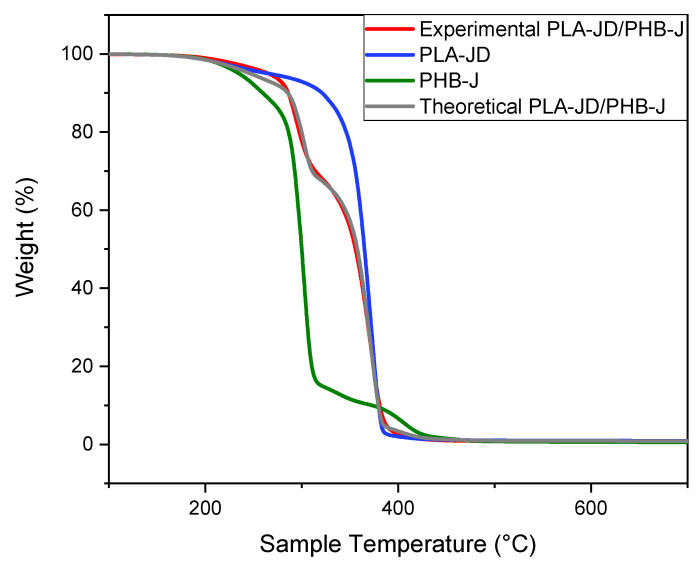
TGA curves of PLA-JD, PHB-J, and experimental and theoretical PLA-JD/PHB-J.

The degradation thermograms of PLA-JD, PHB-J, and experimental PLA-JD/PHB-J blend polymers are shown in Figure 9. The thermograms in Figure 9 show that PHB-J is less stable than PLA-JD. Both polymers begin to decompose close to the processing temperatures. The thermograms of the neat polymers (i.e., PLA-JD and PHB-J) are overlapped to obtain the theoretical degradation curve of the PLA-JD/PHB-J blend, which is presented as theoretical PLA-JD/PHB-J. This thermogram is almost identical to the thermogram of the experimental PLA-JD/PHB-J sample. This shows that there are no interactions between the thermal degradation pathways of the polymers.

**Figure 10:**
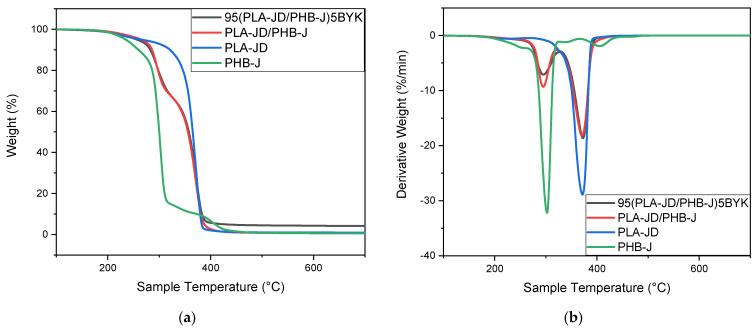
(**a**) TGA and (**b**) derivative thermogravimetric analyses of PLA-JD, PHB-J, PLA-JD/PHB-J, and 95(PLA-JD/PHB-J)5BYK blends.

Figure 10 illustrates the mass loss, as well as the derivative of the mass loss of PLA-JD, PHB-J, and mixtures with and without clay as a function of temperature. It can be seen that the thermal stability decreases when PLA-JD is mixed with PHB-J. When clay is added to the PLA-JD/PHB-J blend, the thermogram of thermal degradation is identical, up to 400 °C, and then a residual mass of about 4% can be observed, corresponding to the inorganic fraction of the organo-modified clay. The presence of a degradation peak at 405 °C on the PHB-J sample can be attributed to the degradation of Joncryl ADR^®^-4468. This peak does not appear on the PLA-JD/PHB-J blend, which explains an improvement in the compatibility between the two polymers with the presence of Joncryl ADR^®^-4468.

**Figure 11:**
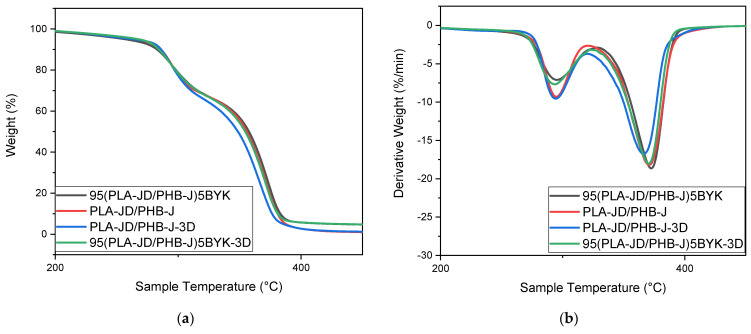
(**a**) TGA and (**b**) derivative thermogravimetric analyses of PLA-JD/PHB-J, and 95(PLA-JD/PHB-J)5BYK injected and printed blends.

Figure 11 illustrates the mass loss, as well as the derivative of the mass loss of PLA-JD/PHB-J and 95(PLA-JD/PHB-J)5BYK injected and 3D-printed samples.

Figure 11 shows that the processing has no influence on the thermal stability of PLA-JD/PHB-J blends with and without clay.

### 3.5. Rheology

**Figure 12:**
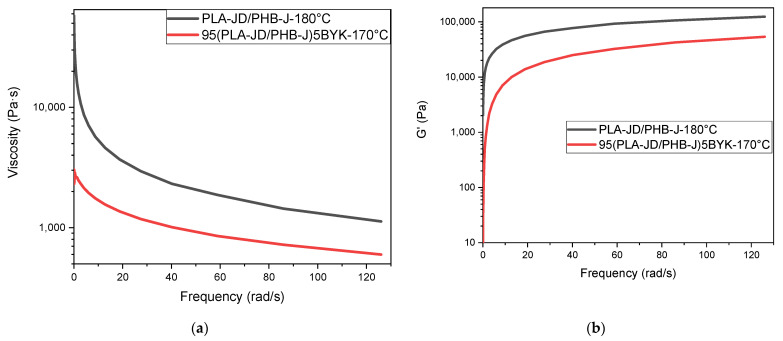
(**a**) Complex viscosity and (**b**) storage modulus in relation to the angular frequency of PLA-JD/PHB-J, and 95(PLA-JD/PHB-J)5BYK blends.

Figure 12 represents the complex viscosity and the storage modulus as a function of frequency for PLA-JD/PHB-J samples, measured at 180 °C, and for 95(PLA-JD/PHB-J)5BYK, measured at 170 °C. The analysis temperature of the sample with OMLS had to be reduced since a reduction in thermal stability was induced by the presence of clay.

Figure 12a shows shear thinning behavior for the two samples tested. Shear-thinning indicates that the blends initially behave as a solid at low frequencies and as a fluid at high frequencies [34]. Figure 12b shows that the storage modulus increases significantly at low frequencies and tends toward a stabilization value above 20 rad/s. Figure 12a,b highlight that the viscosity of the sample and the storage modulus decrease when blends are filled with OMLS, despite a lower analysis temperature. These results may also be associated with the degradation of PHB-J since the clay should act as a reinforcing additive and increase the storage modulus, but the degradation of PHB-J offsets the reinforcing effect of OMLS.

## 4. Conclusions

In this study, we investigated the influence of the addition of organo-modified layered silicate (OMLS) on a PLA/PHB blend in which the polymers were modified by a chain extender with the use of a plasticizer. Two processing routes were carried out: injection molding and 3D printing with FFF.

Regardless of the processing, X-Ray diffraction showed that OMLS was mainly present as intercalated nanostructure and unmodified as Micronic filler. With regard to injection molding, the addition of OMLS to the blend appeared to be detrimental to mechanical properties. Nevertheless, the presence of OMLS improved the compatibility between PLA-JD and PHB-J by the appearance of a single melting peak in DSC. In addition, the reduction in the size of the PHB nodules was observed with AFM and SEM.

Processing by additive manufacturing had no influence on the intercalation of clays. SEM and AFM images revealed a coalescence of the PHB-J phase in the presence of clay, which is ascribed to the degradation of the polymer and the degradation of the organo-modifier of the clay. This phenomenon was confirmed by mechanical (decrease in tensile properties), DSC (appearance of a second melting peak), and rheological (decrease in viscosity and storage modulus) tests.

As a consequence of these results, it was demonstrated that, although using Byko2block 1200 improves the compatibility of the PLA-JD/PHB-J blend, processing by additive manufacturing leads to a strong degradation of the polymer blend, considering its specific thermomechanical history and the degradation of the clay organo-modifier. This degradation can also be accentuated by overly high clay content. For future studies, a decrease in the clay content can be considered in order to decrease polymer degradation. The interest of other kinds of reinforcing ultrafine lamellar fillers with a lower amount of hydroxyl groups such as talc can also be considered in order to limit polymer hydrolysis for 3D printing.

Since degradation seemed also to occur during the processing of the filaments for additive manufacturing, air cooling of the filaments is preferred and can replace water cooling.

## Figures and Tables

**Table 1:** Formulation compositions with weight ratio and code name.

Formulations Composition	Code Name
98 wt% PLA and 2 wt% J	**PLA-J**
99 wt% PHB and 1 wt% J	**PHB-J**
93 wt% PLA, 2 wt% J and 5 wt% DBS	**PLA-JD**
65 wt% PLA, 2 wt% J, 3 wt% DBS and 30 wt% PHB	**PLA-JD/PHB-J**
62 wt% PLA, 2 wt% J, 3 wt% DBS, 28 wt% PHB and 5 wt% BYK	**95(PLA-JD/PHB-J)5BYK**

**Table 2:** Printing parameters for the PLA/PHB blend with and without nanoclay.

Formulation	Nozzle Speed (mm/s)	Printing Temperature (°C)	Printing Speed (mm/s)	Extrusion Multiplier (a.u.)
**PLA-JD/PHB-J-3D**	40	200	60	1.1
**95(PLA-JD/PHB-J)5BYK-3D**	40	195	60	1.05

**Table 3:** Mechanical results of injected and 3D-printed PLA-JD/PHB-J and 95(PLA-JD/PHB-J)5BYK blends.

Blends	Yield Stress (MPa)	Elongation at Yield Stress (%)	Tensile Strength at Break (MPa)	Elongation at Break (%)	Tensile Modulus (MPa)
**PLA-JD**	51.78 ± 1.07	3.33 ± 0.03	19.8 ± 10.2	6 ± 1	2924 ± 189
**PHB-J**	7.58 ± 0.50	3.13 ± 0.38	13.2 ± 0.6	39 ± 8	483 ± 29
**PLA-JD/PHB-J**	9.05 ± 2.05	4.78 ± 0.31	24.5 ± 1.2	505 ± 23	1233 ± 97
**PLA-JD/PHB-J-3D**	25.00 ± 2.10	3.56 ± 0.03	23.1 ± 1.7	219 ± 19	1431 ± 220
**95(PLA-JD/PHB-J)5BYK**	3.45 ± 0.79	4.44 ± 1.14	16.6 ± 0.8	334 ± 12	1355 ± 113
**95(PLA-JD/PHB-J)5BYK-3D**	17.60 ± 0.35	4.23 ± 0.13	21.6 ± 0.6	255 ± 12	954 ± 79

**Table 4:** Temperature and degree of crystallinity measured in the DSC thermograms.

Blends	Tm (°C)	Tg (°C)	Tcc (°C)	X(PLA) (%)	X(PHB) (%)
**PLA-JD**	149	59	112	-	-
**PHB-J**	165	-	-	-	44
**PLA-JD/PHB-J**	146 (PLA-JD) 171 (PHB-J)	30 (PLA-JD) −24 (PHB-J)	92	-	16
**PLA-JD/PHB-J-3D**	142 (PLA-JD) 166 (PHB-J)	38	108	-	30
**95(PLA-JD/PHB-J)5BYK**	146	18	67 81	-	-
**95(PLA-JD/PHB-J)5BYK-3D**	146 (PLA-JD) 158 (PHB-J)	24	81 101	-	-

## Data Availability

Not applicable.
